# The Mediating Role of Emotional Intelligence in the Job Satisfaction and Job Performance of Medical Secretaries: A Cross-Sectional Study

**DOI:** 10.3390/healthcare14182901

**Published:** 2026-09-08

**Authors:** Hanifi Demir

**Affiliations:** Vocational School of Health Services, Bitlis Eren University, 13000 Bitlis, Türkiye; hdemir1@beu.edu.tr

**Keywords:** medical secretary, job satisfaction, self-reported job performance, emotional intelligence, cross-sectional indirect association, health workforce

## Abstract

**Highlights:**

**What are the main findings?**

- Among 134 hospital medical secretaries, adding emotional intelligence to the job-satisfaction model increased the explained variance in self-reported job performance from 7.6% to 30.0%; however, 70.0% of the variance remained unexplained.
- The cross-sectional indirect association through emotional intelligence was statistically supported, but simultaneous measurement cannot establish that job satisfaction precedes emotional intelligence or that emotional intelligence causes better performance.

**What are the implications of the main findings?**

- Organizational practices addressing job satisfaction could be evaluated together with training in emotion regulation, empathic communication, and conflict management rather than being treated as established performance-enhancing interventions.
- Longitudinal, multi-source, and objective-performance studies are needed to compare alternative temporal models and determine whether the observed associations replicate beyond self-report data.

**Abstract:**

**Background/Objectives:** Medical secretaries, who serve as patients’ first point of contact and are frequently exposed to stress arising from illness, waiting, and the hospital environment, remain under-examined in health-workforce research. Previous studies have positioned emotional intelligence in different roles within job-attitude and performance models, including as an antecedent of job satisfaction. Because cross-sectional self-report data cannot establish temporal ordering or causal mediation, the present study examined whether emotional intelligence statistically accounts for part of the cross-sectional association between medical secretaries’ job satisfaction and self-reported job performance. **Methods:** A cross-sectional study was conducted with medical secretaries from two public hospitals in Bitlis Province, Türkiye (April–May 2025). Of 176 eligible employees invited, 134 complete questionnaires were analyzed (response rate: 76.1%). Emotional intelligence was assessed with the Wong and Law Emotional Intelligence Scale, job satisfaction with the five-item short form of the Brayfield–Rothe scale, and self-reported job performance with a four-item scale. Group comparisons, Pearson correlations, linear regression, a 10,000-resample bootstrap indirect-association analysis, a reverse-model sensitivity analysis, and a Harman single-factor diagnostic were performed. **Results:** Job satisfaction was positively associated with self-reported job performance (r = 0.275; *p* = 0.001) and emotional intelligence (r = 0.341; *p* < 0.001), while emotional intelligence showed a stronger association with performance (r = 0.539; *p* < 0.001). The conditional direct association of job satisfaction with performance was not statistically significant after emotional intelligence entered the model (β = 0.103; *p* = 0.187); the cross-sectional indirect association through emotional intelligence was 0.114 (95% bootstrap CI: 0.042–0.204). Explained variance increased from 7.6% to 30.0%, leaving 70.0% unexplained. In the reverse specification (emotional intelligence → job satisfaction → performance), the indirect association was not statistically supported (ab = 0.009; 95% bootstrap CI: −0.005 to 0.025). Harman’s first unrotated component accounted for 38.7% of item variance. **Conclusions:** Emotional intelligence accounted statistically for a meaningful but incomplete portion of the job satisfaction–performance association in this sample. The results are compatible with the proposed indirect pathway but do not establish temporal order or causality. Replication using longitudinal designs, multiple data sources, objective performance indicators, and additional occupational factors such as emotional labor, burnout, workload, and organizational support is warranted.

## 1. Introduction

In healthcare institutions, medical secretaries are health personnel who constitute an important component of the healthcare team throughout the process from patients’ first admission to their discharge. By carrying out tasks such as appointment scheduling, patient admission and registration, file and document management, patient guidance, and the coordination of physician–patient communication, medical secretaries play a critical role in the functioning of service delivery and constitute one of the groups most exposed to social interaction in healthcare institutions. As they form the patient’s first point of contact with the healthcare organization, they play an important role in ensuring effective and efficient communication among patients, health professionals, and managers, and in helping healthcare institutions achieve their quality objectives [1,2,3]. The health personnel with whom patients and their relatives first communicate in healthcare institutions are usually medical secretaries; they therefore hold a key position in the orderly and harmonious functioning of the health system [4].

Job satisfaction is a global evaluative attitude toward one’s job and reflects how employees appraise their work and its conditions [5]. Although satisfied employees may report greater willingness to remain engaged with their work, the relationship with performance is not deterministic. A comprehensive meta-analysis estimated a corrected mean correlation of approximately 0.30 between job satisfaction and job performance, with variation across occupations and job complexity [6]. Research relevant to medical secretaries and hospital employees also indicates that supervisor and co-worker support, workload planning, and other organizational conditions are related to performance and satisfaction [7,8,9]. Job satisfaction should therefore be regarded as one performance-related correlate among several rather than as a sufficient explanation of performance.

Job performance is a multidimensional construct describing behaviors and outputs that contribute to organizational goals. It is commonly differentiated into task performance, which concerns the execution of core job duties, and contextual performance, which includes discretionary behaviors that support the social and organizational environment. For medical secretaries, performance may encompass timely and accurate completion of administrative tasks, coordination of patient flow and documentation, problem solving, and effective communication with patients, relatives, clinicians, and managers [10]. Performance can be measured through self-report, supervisor or peer ratings, and administrative or objective indicators; these approaches capture overlapping but not identical aspects of work performance.

Emotional intelligence (EI) was originally defined as the capacity to monitor one’s own and others’ emotions, discriminate among them, and use emotional information to guide thinking and action [11]. The EI literature includes distinct conceptual traditions, including performance-based ability models, self-report/trait-oriented approaches, and mixed models; instruments derived from these traditions are not interchangeable because they assess different aspects of emotional functioning [12]. The present study uses the Wong and Law framework and conceptualizes the WLEIS score as self-reported perceived emotional competence in four domains: self-emotion appraisal, others’ emotion appraisal, use of emotion, and regulation of emotion [13]. In this interpretation, WLEIS is relatively trait-like but remains a self-evaluation of one’s typical emotional functioning rather than an objective performance test or a momentary emotional state. The study therefore does not assume that job satisfaction rapidly changes an underlying stable EI trait. Instead, the focal cross-sectional model asks whether perceived EI-related competencies, and potentially their context-dependent deployment or self-appraisal at work, statistically account for part of the covariance between job satisfaction and self-reported performance. The four domains are directly relevant to frontline healthcare work: self-emotion appraisal may support self-monitoring under pressure; appraisal of others’ emotions may facilitate recognition of anxiety or anger; use of emotion may support goal-directed effort; and emotion regulation may help sustain professional communication during conflict or uncertainty. Consistent with this rationale, EI has been associated with caring behavior, well-being, work attitudes, and performance-related outcomes [13,14,15].

Emotional labor provides an occupational framework linking these constructs. Medical secretaries are expected to remain calm, courteous, and helpful even when patients or relatives are anxious, angry, or distressed, creating a requirement to manage felt and displayed emotion [16]. Grandey’s emotion-regulation perspective conceptualizes such regulation as a central mechanism of emotional labor, while healthcare models have linked emotional labor with job satisfaction and performance [16,17]. Within this framework, greater job satisfaction may covary with stronger motivational engagement and a greater willingness or perceived capacity to deploy adaptive emotion-regulation strategies, whereas dissatisfaction, strain, or burnout may accompany emotional depletion and more effortful surface regulation. WLEIS dimensions capture self-perceived competencies that could support the appraisal and regulation required by these encounters. This makes a satisfaction-EI-performance ordering theoretically plausible as a cross-sectional representation of work attitude, perceived emotional functioning, and perceived performance, but it does not establish that satisfaction causes EI. The reverse ordering, in which EI precedes satisfaction, is also theoretically plausible and is examined as a sensitivity model. EI is also only one of several possible explanatory factors: emotional labor, occupational stress, burnout, workload, organizational support, job crafting, staffing conditions, and role demands may influence employee well-being and performance [8,9,10,16,17,18,19,20,21,22].

The direction of the focal model therefore requires explicit qualification. Previous healthcare research has tested different configurations; for example, Chauhan et al. positioned job satisfaction between emotional intelligence and nurses’ job performance, while other studies have examined conflict management, occupational stress, moral intelligence, clinical competence, and emotional labor in related pathways [17,18,19,23,24,25,26,27]. The focal ordering represents the study’s original hypothesis model and tests whether perceived EI-related competencies statistically account for part of the satisfaction–performance association in medical secretaries; it is not a developmental claim that job satisfaction changes a stable emotional capacity. A plausible occupational interpretation is that favorable work attitudes may coexist with greater engagement in, or more favorable self-appraisal of, the emotional-appraisal and regulation processes required during demanding patient encounters, and those same perceived competencies may covary with more effective day-to-day functioning. Because the variables were measured concurrently, however, this mechanism is not temporally identified. Importantly, H4 is not inferred from the conditional direct association (c′) becoming non-significant. The inferential criterion is whether the bootstrap confidence interval for the indirect product ab excludes zero; neither a significant total association nor a non-significant c′ is required for a statistical indirect effect [28,29]. Accordingly, c′ is reported descriptively and is not interpreted as proof of ‘full mediation’. Even a statistically supported ab term cannot demonstrate temporal sequence or causal mediation in cross-sectional data [30]. A post hoc sensitivity analysis therefore tested the theoretically plausible reverse specification (EIS → JSS → JPS). The following hypotheses were evaluated: (H1) job satisfaction is positively associated with self-reported job performance; (H2) job satisfaction is positively associated with emotional intelligence; (H3) emotional intelligence is positively associated with self-reported job performance; and (H4) the cross-sectional indirect association between job satisfaction and self-reported job performance through emotional intelligence is statistically different from zero. The sociodemographic comparisons were secondary and exploratory.

The contribution of the present study therefore does not lie in proposing that job satisfaction, emotional intelligence, and performance are newly related constructs; those pairwise associations have been widely studied in healthcare and service research. The intended contribution is a more specific boundary-condition and model-clarification question in an understudied frontline administrative role. Medical secretarial work combines accuracy-sensitive administrative coordination with continuous patient-facing emotional display requirements, often without the clinical authority or professional autonomy available to nurses or physicians. This combination may make the joint pattern of job attitudes, perceived emotional competence, and perceived performance different from that observed in purely administrative, managerial, or clinical samples. Within the medical-secretary literature reviewed here, prior studies have mainly examined supervisor or co-worker support, emotional contagion, workload, job stress, and communication with difficult patients [2,3,4,7,9,20], rather than jointly testing satisfaction, EI, and performance. The present study therefore (i) evaluates whether EI adds cross-sectional explanatory information beyond job satisfaction for self-reported performance, (ii) compares the focal ordering with a theoretically plausible reverse indirect model, (iii) examines the measurement structure and same-source measurement concerns in the study sample, and (iv) considers emotional labor as the occupational mechanism that makes this configuration theoretically relevant. Because the design is cross-sectional, these contributions are model-clarifying and hypothesis-generating rather than causal.

## 2. Materials and Methods

### 2.1. Study Design and Setting

This cross-sectional, correlational study was conducted in two public hospitals in Bitlis Province, Türkiye, between April and May 2025. The article was prepared in line with the Strengthening the Reporting of Observational Studies in Epidemiology (STROBE) recommendations for cross-sectional studies.

### 2.2. Participants

The study population consisted of all 176 employees listed by the two participating hospitals as medical secretaries during the data-collection period. Eligibility required current employment in a medical-secretary role at one of the two hospitals and voluntary informed consent. No minimum employment duration, specific hospital unit, or restricted set of secretarial duties was prespecified; the intention was to enumerate the full medical-secretary workforce rather than a selected task subgroup. All 176 eligible employees were invited without sampling, and only fully completed questionnaires were included in the analysis. A total of 134 questionnaires were analyzable, yielding an overall response rate of 76.1%. No missing item responses or out-of-range values were found among these questionnaires. Administrative workforce counts allowed response rates to be examined by hospital: 83 of 117 eligible employees participated in one hospital (70.9%) and 51 of 59 in the other (86.4%); the response proportions differed (χ^2^(1) = 5.19; *p* = 0.023). Age, gender, tenure, education, and other characteristics of the 42 non-participating employees were not available, so a broader participant-versus-nonparticipant comparison could not be performed and non-response bias cannot be excluded.

### 2.3. Data Collection Instruments

- Sociodemographic form

Prepared by the researcher, this form collected information on participants’ gender, marital status, age, education level, total length of service as a medical secretary, and the hospital in which they worked.

- Emotional Intelligence Scale (EIS)

The 16-item WLEIS developed by Wong and Law was used to assess participants’ self-reported emotional intelligence [13]. The Turkish wording administered in the present questionnaire followed the version documented by Uslu [31], where a WLEIS text supplied by Wong was translated into Turkish and reliability/validity analyses were reported in a Turkish working sample. Atilla et al. [32] is retained as an earlier empirical application of WLEIS among Turkish hospital employees, but it is not described here as the formal Turkish adaptation source. Because no independent, stand-alone cross-cultural validation study establishing a definitive Turkish WLEIS version was identified, the administered form is described here as a previously used Turkish translation/application rather than as a formally validated national adaptation. The scale comprises four subdimensions (self-emotion appraisal, others’ emotion appraisal, use of emotion, and regulation of emotion); items are rated on a 5-point Likert scale and total scores range from 16 to 80. WLEIS was selected because it is brief, workplace-oriented, and directly covers the four emotion-related domains central to the study model while limiting response burden during hospital work. It is a self-report measure of perceived/typical emotional functioning rather than a performance-based test of emotional ability. In this sample, Cronbach’s alpha was 0.93 for the total scale and 0.91, 0.81, 0.84, and 0.82 for the four subdimensions, respectively. Sample-specific construct-validity checks are reported below.

- Job Satisfaction Scale (JSS)

Job satisfaction was measured with the 5-item short form of the scale originally developed by Brayfield and Rothe [33] and abbreviated by Judge et al. [34]; the Turkish adaptation study was conducted by Başol and Çömlekçi [35]. Items are rated on a 5-point Likert scale; the two negatively worded items were retained and reverse-scored, and total scores range from 5 to 25. This brief global measure was selected because the study focused on overall job satisfaction rather than satisfaction with separate job facets and because its short format reduced respondent burden in conjunction with the EI and performance measures. Cronbach’s alpha in this study was 0.85.

- Job Performance Scale (JPS)

Job performance was assessed with a 4-item measure derived from Kirkman and Rosen [36] and administered using the individual self-report wording previously used in Turkish organizational research by Çöl [37]. In practical terms, the construct was shifted from a team/managerial frame to first-person judgments of the respondent’s own task completion, goal attainment, service quality, and problem solving; no new performance domains were added. The same general four-item instrument has subsequently been used as a self-reported ‘performance perception’ measure in Turkish employees, with Uslu et al. reporting Cronbach’s alpha = 0.794 in 371 call-center workers [38]. The present study nevertheless did not conduct a pre-study content-validity panel, cognitive interviewing, supervisor criterion validation, or objective-performance validation for medical secretaries. Therefore, the measure is explicitly interpreted as perceived self-reported job performance, not as an interchangeable proxy for the original manager-rated team construct or for objective productivity. In the current sample, the four items showed Cronbach’s alpha = 0.88 and satisfactory loadings/AVE in the measurement model reported below; these within-sample findings support internal structure but do not establish criterion validity or equivalence across measurement sources. Objective indicators were not specified in the original protocol, and the anonymous questionnaires could not be linked to administrative or supervisor records; registration/documentation error rates, processing times, patient-flow metrics, and supervisor ratings were therefore unavailable.

Instrument selection involved a trade-off between construct coverage, measurement source, and feasibility. Other EI instruments include performance-based measures such as the Mayer-Salovey-Caruso Emotional Intelligence Test and trait-oriented or mixed-model questionnaires such as the TEIQue and EQ-i [12]. Job satisfaction can be assessed with longer multidimensional instruments, including Spector’s Job Satisfaction Survey [39] and the Minnesota Satisfaction Questionnaire [40]. Broader work-performance instruments, such as the Individual Work Performance Questionnaire, assess multiple performance domains [41], while supervisor/peer ratings and objective administrative indicators can provide non-self-report criteria. The brief measures used here minimized survey burden, but the modified self-report use of the performance scale and the reliance on a WLEIS total score required additional sample-specific measurement checks and are treated as limitations rather than assumed to be fully resolved by prior use.

### 2.4. Data Analysis

Descriptive statistics, independent-samples *t* tests, one-way analysis of variance (ANOVA) with Bonferroni-corrected pairwise comparisons, Pearson correlation analysis, and linear regression were used. No a priori power analysis was conducted. Instead, a sensitivity calculation was performed using the noncentral F distribution in Python 3.12/SciPy under the fixed-model linear multiple-regression, R^2^-deviation-from-zero framework: *N* = 134, alpha = 0.05, the omnibus F test, target power = 0.80, with one predictor for the single-predictor model and two predictors for the joint model. These parameters yielded minimum detectable effect sizes of f^2^ = 0.059 and 0.074, respectively, consistent with the regression power-analysis framework described by Faul et al. [42] Analyses were performed using IBM SPSS 26 Statistics (IBM Corp., Armonk, NY, USA); descriptive and inferential results and percentile-bootstrap indirect-association estimates based on 10,000 resamples were independently reproduced and verified from the raw data in Python 3.12 (NumPy and SciPy). Distributional adequacy of the observed scale totals was initially evaluated using skewness and kurtosis coefficients; the maximum absolute skewness was 1.59 and maximum absolute kurtosis was 3.03, within commonly used descriptive thresholds [43,44]. The focal cross-sectional indirect association was estimated using regression-based path analysis with 10,000 percentile-bootstrap resamples [28,29]. Statistical support for H4 was defined by a 95% bootstrap confidence interval for the indirect product ab that did not include zero; the significance or non-significance of c′ was not used as the criterion for an indirect effect [28,29]. Because mediation is inherently temporal, the result was interpreted only as a cross-sectional statistical indirect association [30]. A post hoc reverse model (EIS → JSS → JPS) was estimated with the same bootstrap procedure.

Additional measurement and diagnostic analyses were conducted in response to the limitations of the self-report design. First, a correlated six-factor confirmatory measurement model was estimated by maximum-likelihood covariance fitting using the 25 substantive items: the four WLEIS facets, job satisfaction, and self-reported job performance. Because the items used five response categories and the sample was modest (*N* = 134), this CFA was treated as a pragmatic, sample-specific structural check rather than a definitive psychometric validation. Model fit was summarized with chi-square, CFI, TLI, RMSEA, and SRMR; standardized factor loadings, composite reliability (CR), average variance extracted (AVE), the Fornell–Larcker criterion, and the heterotrait–monotrait ratio (HTMT) were used as indicators of convergent and discriminant validity [45,46,47]. Because the primary regression analyses used the WLEIS total score, a complementary one-factor model using the four WLEIS subscale scores as indicators of a general EI summary was also evaluated. Second, regression assumptions were evaluated using residual and partial-residual inspection, Ramsey’s RESET for functional form, Q-Q/residual normality checks, the Breusch–Pagan test for heteroscedasticity, variance inflation factors (VIFs) and tolerance for multicollinearity, and Cook’s distance, leverage, and externally studentized residuals for influential observations. HC3 heteroscedasticity-consistent standard errors and leave-one-out estimates were used as robustness checks. Third, because 15 omnibus demographic comparisons were performed (five characteristics across three outcomes), their *p* values were treated as exploratory; a post hoc Holm family-wise correction was used as a multiplicity sensitivity analysis in addition to the within-education Bonferroni pairwise comparisons.

Because all substantive variables were obtained from the same respondents in one survey, procedural and statistical common-method considerations were also examined. The questionnaire was anonymous and voluntary, used standardized instructions, and presented EI, job satisfaction, and job performance in separate sections, which reduced direct item-context cues. However, there was no temporal separation, source separation, marker variable, or dedicated social-desirability scale. A post hoc Harman single-factor diagnostic was therefore conducted using all 25 substantive items in an unrotated principal-components analysis. A dominant single factor or a first component accounting for the majority of total item variance would raise concern, but Harman’s procedure is only a coarse diagnostic and cannot rule out common method variance [48]. Because no dedicated social-desirability measure was collected, the magnitude of socially desirable responding could not be estimated or statistically adjusted. All 134 complete questionnaires were analyzed, and two-tailed *p* < 0.05 was used for the primary association analyses.

### 2.5. Ethical Considerations

Approval for the study was obtained from the Social and Human Sciences Research Ethics Committee of Bitlis Eren University (protocol code 2025/01-3, E.6847; date of approval 5 February 2025). Institutional permissions were obtained from the hospitals concerned. Participants were informed about the purpose of the study, participation was based on voluntariness and anonymity, and informed consent was obtained from all participants. All procedures were carried out in accordance with the Declaration of Helsinki.

## 3. Results

### 3.1. Participant Characteristics

The study was completed with the participation of 134 medical secretaries. Of the participants, 56.0% were under 30 years of age, 66.4% were male, and 52.2% were married. The majority held an associate degree (62.7%), and 64.2% had four years or less of professional experience (Table 1). The participant distribution by hospital was 83 (61.9%) and 51 (38.1%). In post hoc Welch comparisons among respondents, mean EIS, JSS, and JPS scores did not differ significantly between the two hospitals (*p* = 0.794, *p* = 0.828, and *p* = 0.396, respectively); nevertheless, the hospital-specific response rates differed as reported in Section 2.2, so institution-related non-response cannot be excluded.

### 3.2. Comparison of Scale Scores by Participant Characteristics

Comparisons of scale scores by participant characteristics are presented in Table 2 using nominal, unadjusted omnibus *p* values because these analyses were secondary and exploratory. Emotional intelligence and self-reported job performance scores did not differ significantly by age, whereas participants aged 30 and over had higher job satisfaction scores (t(132) = −2.30; *p* = 0.023; Cohen’s d = 0.40). Women had higher emotional intelligence (t(132) = −2.67; *p* = 0.009; Cohen’s d = 0.49) and self-reported job performance scores (t(132) = −2.65; *p* = 0.009; Cohen’s d = 0.48), while job satisfaction did not differ by gender. No nominal differences were found by marital status or work experience. Emotional intelligence differed by education level (F(2, 131) = 5.63; *p* = 0.004; η^2^ = 0.079), and Bonferroni-corrected pairwise comparisons indicated higher scores among associate- and bachelor’s-degree holders than among those with high-school education or below. However, when the 15 omnibus demographic tests were considered together using a post hoc Holm correction, none remained significant at the 0.05 family-wise level; the smallest adjusted *p* value was 0.067 for the education-EI comparison. Accordingly, all demographic findings should be interpreted as hypothesis-generating rather than confirmatory.

### 3.3. Measurement-Model Checks

The correlated six-factor measurement model (four WLEIS facets, JSS, and JPS) showed an adequate but not perfect fit to the item-level data: χ^2^(260) = 437.95, *p* < 0.001; CFI = 0.915; TLI = 0.902; RMSEA = 0.072; SRMR = 0.065. Standardized loadings ranged from 0.479 to 0.933. Composite reliability ranged from 0.815 to 0.915 and AVE from 0.530 to 0.731 across the six factors. For each factor, the square root of AVE exceeded its correlations with the other factors, and HTMT values ranged from 0.236 to 0.783, below the commonly used 0.85 criterion [46,47]. These results provide sample-specific support for convergent and discriminant validity while also indicating that measurement fit is not flawless. Because WLEIS is four-dimensional, a complementary one-factor CFA using its four subscale scores as indicators of a general EI summary was examined to justify the total score used in the regression analyses. This model fitted the four subscale scores closely (χ^2^(2) = 0.52, *p* = 0.771; CFI = 1.000; RMSEA < 0.001; SRMR = 0.011), with standardized loadings from 0.693 to 0.878. Thus, the total WLEIS score was retained as a parsimonious higher-order summary for the focal association analyses, while the four facets remain conceptually distinct. Given the modest sample and self-report format, these analyses should be interpreted as within-sample psychometric checks rather than a substitute for independent scale validation.

### 3.4. Correlations Among the Variables

Job satisfaction was positively correlated with self-reported job performance (r = 0.275; *p* = 0.001) and emotional intelligence (r = 0.341; *p* < 0.001); emotional intelligence showed a stronger positive correlation with self-reported performance (r = 0.539; *p* < 0.001) (Table 3). These cross-sectional correlations do not indicate temporal order or causality.

### 3.5. Cross-Sectional Indirect-Association Analysis

The regression models are summarized in Table 4. Job satisfaction was positively associated with self-reported job performance (B = 0.183; SE = 0.056; β = 0.275; *p* = 0.001; R^2^ = 0.076), consistent with H1. Job satisfaction was also positively associated with emotional intelligence (B = 0.849; SE = 0.204; β = 0.341; *p* < 0.001; R^2^ = 0.116), consistent with H2. In the joint model, the conditional direct association of job satisfaction with performance was not statistically significant (B = 0.069; SE = 0.052; β = 0.103; *p* = 0.187), whereas emotional intelligence remained positively associated with self-reported performance (B = 0.134; SE = 0.021; β = 0.504; *p* < 0.001), consistent with H3. The joint model explained 30.0% of the variance in self-reported job performance (R^2^ = 0.300; adjusted R^2^ = 0.289), leaving 70.0% unexplained. The increase in R^2^ from 7.6% to 30.0% is interpreted only as incremental explanatory information from adding EI; it is not evidence of mediation or of an indirect mechanism.

**Figure 1:**
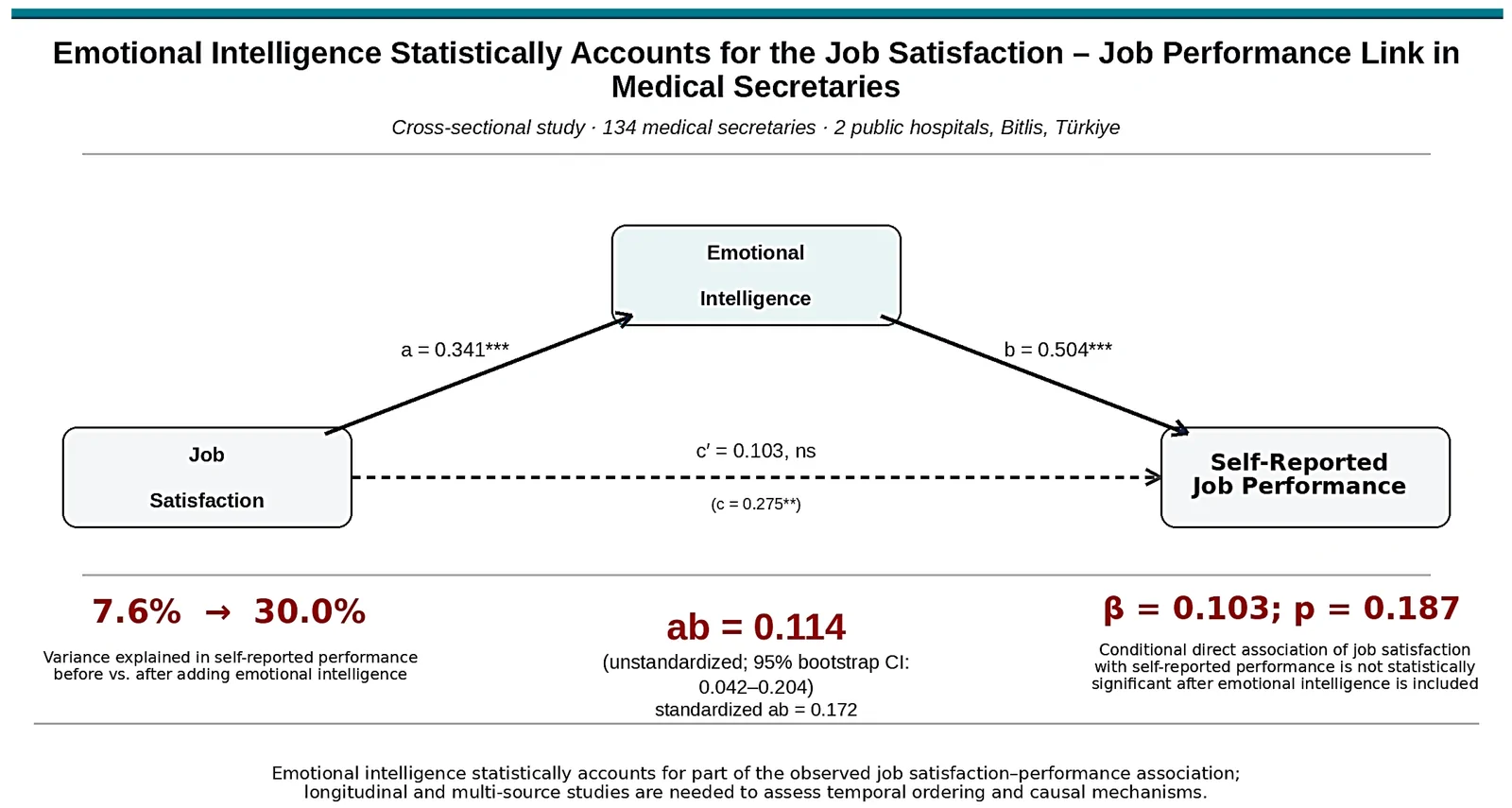
Cross-sectional indirect-association model linking job satisfaction, emotional intelligence, and self-reported job performance. Path coefficients (a, b, c, and c′) are standardized; c′ denotes the conditional direct path after controlling for emotional intelligence, and c denotes the total association. The dashed arrow represents the statistically non-significant conditional direct path (c′). ** *p* < 0.01; *** *p* < 0.001; ns, not significant. Note: bootstrap ab and its 95% confidence interval are reported in unstandardized units; standardized ab = 0.172.

The estimated cross-sectional indirect association of job satisfaction with self-reported job performance through emotional intelligence was 0.114 (95% bootstrap CI: 0.042–0.204; 10,000 resamples; standardized indirect association = 0.172), consistent with H4 at the statistical level (Figure 1). The bootstrap interval for ab, rather than the change in R^2^ or the non-significance of c′, is the inferential basis for the indirect-association result. Because all three variables were measured at the same time, this finding is compatible with, but does not establish, the proposed temporal ordering.

### 3.6. Regression, Common-Method, and Alternative-Model Sensitivity Analyses

Regression diagnostics indicated low multicollinearity in the joint model (VIF = 1.13 for both predictors; tolerance = 0.884). Ramsey RESET did not indicate functional-form misspecification for the JSS → EIS path model (*p* = 0.248) or the joint JPS model (*p* = 0.726). Residual normality was imperfect (Shapiro–Wilk *p* < 0.001 for the path models), and Breusch–Pagan tests indicated heteroscedasticity for the JSS → EIS model (*p* = 0.046) and especially for the joint JPS model (*p* < 0.001). In the joint model, maximum leverage was 0.105; five observations exceeded the conventional 3(k + 1)/*N* screening threshold, but no observation had Cook’s distance ≥ 1 (maximum = 0.407), and three observations had externally studentized residuals with absolute values > 3. HC3 heteroscedasticity-consistent standard errors did not change the inferential pattern: JSS remained associated with EIS (B = 0.849; *p* < 0.001), EIS remained associated with JPS in the joint model (B = 0.134; *p* = 0.001), and the conditional JSS-JPS coefficient remained non-significant (B = 0.069; *p* = 0.206). In leave-one-out analyses, the product ab remained positive in every iteration and ranged from 0.099 to 0.134. Adding hospital as a post hoc covariate likewise left the EI coefficient essentially unchanged (B = 0.135; *p* < 0.001), while hospital was not independently associated with JPS (*p* = 0.233; model R^2^ = 0.307). These checks support the stability of the coefficient pattern but do not remove assumption limitations, measurement limitations, or hospital-level non-response concerns. Harman’s single-factor diagnostic showed six components with eigenvalues greater than 1; the first unrotated component accounted for 38.7% of total variance across the 25 substantive items. Thus, one general factor did not account for the majority of item variance, but this diagnostic neither quantifies nor excludes common method variance [48]. No dedicated social-desirability measure was included, so social-desirability bias could not be tested or adjusted. In the post hoc reverse specification (EIS → JSS → JPS), the unstandardized indirect association was 0.009 and its 95% bootstrap CI included zero (−0.005 to 0.025; 10,000 resamples). The reverse indirect association was therefore not statistically supported in this sample, but the comparison cannot establish temporal priority because all measures were concurrent [30].

## 4. Discussion

This study examined whether emotional intelligence statistically accounts for part of the cross-sectional association between medical secretaries’ job satisfaction and self-reported job performance. Job satisfaction was associated with performance when examined alone, but its conditional direct association was not statistically significant after EI was included, while EI remained positively associated with performance and the bootstrap interval for ab excluded zero. The statistical basis for H4 is the bootstrapped indirect product ab, not the loss of significance of c′ and not the increase in R^2^ [28,29]. The rise in explained variance from 7.6% to 30.0% indicates that EI contributes incremental explanatory information beyond job satisfaction, whereas the bootstrap result addresses the indirect statistical association; these are distinct findings. Current indirect-effect methods do not require c′ to become non-significant, and simultaneous measurement means that the temporal assumptions required for causal mediation remain untested [30]. The joint model still left 70.0% of self-reported performance variance unexplained. The satisfaction–performance correlation (r = 0.275) is close to the meta-analytic mean reported in broader occupational research [6], while the incremental information associated with EI is consistent with meta-analytic evidence linking EI to work performance [49].

The theoretical contribution is best understood as a boundary-condition and occupational-process contribution rather than the discovery of new pairwise relationships. Medical secretaries combine accuracy-sensitive administrative duties with intensive emotional labor at the patient-service interface. They are expected to manage queues, documentation, appointments, and communication while maintaining socially appropriate emotional displays in encounters involving anxiety, anger, uncertainty, and time pressure [1,3,4,16]. In Grandey’s framework, emotion regulation is the mechanism through which emotional labor is enacted [16]. WLEIS facets map onto capacities that may support this process: recognizing one’s own activation, reading others’ affective cues, maintaining goal-directed motivation, and regulating reactions. Job satisfaction may be associated with greater work engagement and a greater perceived capacity or willingness to deploy these strategies, whereas strain or dissatisfaction may coexist with emotional depletion; however, these mechanisms were not directly measured and therefore remain theoretical explanations rather than demonstrated mediators. The distinctive value of the medical-secretary context is that these emotional-regulation demands occur alongside administrative performance requirements but outside a primarily clinical role. This combination may make perceived EI particularly relevant to how favorable work attitudes and perceived performance covary. Job Demands-Resources theory simultaneously implies that workload, burnout, staffing, role clarity, and organizational support may shape the same relationships [21], so EI should be regarded as one possible personal resource within a broader system of demands and resources.

Comparison with prior studies also shows why model direction should not be treated as settled. Chauhan et al. [26] studied nurses and positioned job satisfaction downstream of EI and upstream of performance, the reverse of the focal ordering tested here. Soriano-Vazquez et al. [23] likewise treated EI as an antecedent but introduced conflict management as the intervening mechanism for job satisfaction. Hwang and Park [19] embedded emotional labor and job satisfaction in a structural model of nurses’ performance, while other healthcare studies have examined moral intelligence and occupational stress [17,18] or clinical competence [24] as intervening processes. Service-sector studies have linked EI with employee adaptability, satisfaction, and performance in broader frontline samples [50,51]. These studies differ not only in occupational group but also in the direction assigned to EI and in the mechanisms placed between attitudes and performance. The present post hoc reverse EIS → JSS → JPS indirect association was small and its bootstrap interval included zero, whereas the focal JSS → EIS → JPS indirect association excluded zero. This is sample-specific statistical evidence favoring the focal configuration within these concurrent data, not evidence that the focal temporal ordering is true or that the reverse model is false. The cascading model and other EI theories continue to support a relatively stable EI antecedent interpretation [52]. Longitudinal, multi-wave, and multi-source studies are required to distinguish these alternatives.

The sociodemographic findings were secondary and exploratory. At the nominal *p*-value level, participants aged 30 years or older reported higher job satisfaction, women reported higher EI and self-reported performance, and associate- and bachelor’s-degree holders reported higher EI than those with high-school education or below. However, none of the 15 omnibus demographic comparisons remained significant after the post hoc Holm family-wise correction, so these patterns should not be treated as robust subgroup effects. The education pattern remains useful as a hypothesis for future work. More highly educated employees may have had greater exposure to communication, psychology, management, or interpersonal-skills content, or may have been assigned to roles with greater administrative complexity, responsibility, patient contact, or conflict-management demands. Conversely, higher-level positions may impose greater interpersonal and performance expectations and may encourage stronger self-monitoring or more favorable self-assessment. Selection effects are also possible if individuals with stronger pre-existing emotional skills are more likely to pursue further education or be allocated to demanding roles. Because job title, task mix, training history, position level, and patient-contact intensity were not measured, the nominal education difference cannot be attributed to education itself. The same caution applies to the nominal age and gender patterns.

From a practical perspective, the findings suggest areas that could be evaluated rather than interventions whose effectiveness is established by this study. Healthcare organizations may consider piloting or testing programs that combine attention to job-satisfaction-related working conditions with training in emotional awareness, emotion regulation, empathic communication, conflict management, and stress coping. A systematic review and meta-analysis has reported improvements in emotional-intelligence scores after training among healthcare workers [53], but the present cross-sectional data do not show that such training would improve medical secretaries’ job performance. Likewise, medical-secretarial curricula could consider including emotion management and professional communication alongside technical skills, with effectiveness assessed prospectively. Hiring, promotion, or performance decisions should not be based solely on self-reported emotional-intelligence scores.

### 4.1. Strengths and Limitations

The strengths of the study include the attempt to reach the entire defined target population, a 76.1% overall response rate, analysis of all 134 complete questionnaires without post hoc exclusions, good internal consistency, sample-specific measurement-model checks, bootstrap estimation of the indirect association, and sensitivity analyses for model direction, regression assumptions, hospital, and common method variance. Several limitations remain. First, the cross-sectional design does not permit inference about temporal ordering or causality. The data cannot distinguish among the focal JSS → EI → performance ordering, the theoretically plausible EI → JSS → performance ordering, or a model in which EI is a common antecedent of both satisfaction and performance; the reverse-model sensitivity analysis does not solve this temporal-identification problem [30,52]. Second, all substantive variables were self-reported by the same participants at the same time. Shared response tendencies, consistency motifs, acquiescence, and socially desirable self-presentation may have strengthened the positive associations. Anonymity and separation of constructs into questionnaire sections were procedural safeguards, and Harman’s first component accounted for 38.7% rather than a majority of item variance, but Harman’s test is limited and no social-desirability scale or marker variable was available [48]. Third, the job-performance measure was used as an individual self-report even though its source instrument was originally framed at team level and rated managerially. The present study used previously published Turkish individual-level wording rather than creating new item content, and prior Turkish self-report applications provide some reliability evidence [37,38]; nevertheless, the change in level and source may affect construct and criterion validity and was not formally validated for medical-secretary work before data collection. The favorable within-sample factor loadings, reliability, AVE, and discriminant-validity results do not demonstrate equivalence with manager-rated or objective performance. Fourth, WLEIS is a four-dimensional self-report instrument. The higher-order subscale analysis supported use of a total EI summary in this sample, but self-reported EI does not necessarily converge with performance-based ability measures and the sample-specific CFA does not replace independent Turkish validation [12]. Fifth, the 42 non-participating employees could not be compared with participants on demographic or occupational characteristics. Response rates differed by hospital (70.9% vs. 86.4%), creating a plausible source of non-response bias even though respondent scale means did not differ significantly between hospitals. Sixth, organizational culture, staffing, workload, management practices, and other hospital-level conditions were not measured, so the influence of the two institutions cannot be separated from individual-level relationships. Seventh, the joint model explained 30.0% of self-reported performance variance, leaving 70.0% unexplained; emotional labor, burnout, workload, organizational support, role clarity, employment conditions, task complexity, patient-contact intensity, and digital/administrative competence are among the additional factors that may contribute [8,9,10,17,18,19,20,21,22]. Finally, the two public hospitals were located in a single province, and the sample size limited more complex multilevel, moderated, and subgroup models.

### 4.2. Directions for Future Research

Future research should address temporal ordering, measurement validity, and institutional context simultaneously. Multi-wave designs should compare competing sequences, for example EI at Time 1 → job satisfaction at Time 2 → performance at Time 3 versus job satisfaction at Time 1 → emotion-regulation-related processes at Time 2 → performance at Time 3. Performance should be triangulated using supervisor or co-worker ratings and objective indicators such as documentation accuracy, registration or appointment errors, processing time, service throughput, absenteeism, and other role-appropriate administrative metrics. Future studies should use a performance measure validated specifically for individual medical-secretary work or conduct a formal content and criterion validation when adapting an existing measure. Same-source surveys should incorporate stronger method-bias controls, including temporal or source separation where feasible, a validated social-desirability scale or marker variable, and latent-method approaches in adequately powered samples. Larger multicenter studies should test measurement invariance of the WLEIS and performance measures across hospitals and regions and should model hospital-level organizational culture, workload, staffing, and managerial support. Emotional labor, burnout, role clarity, patient-contact intensity, job crafting, employment conditions, and digital/administrative competence should be incorporated to investigate the currently unexplained 70.0% of performance variance. Ability-based EI measures could also be used alongside WLEIS to distinguish perceived emotional competence from performance-based emotional ability. Finally, prospective training or organizational-intervention studies with pre-specified objective and rated outcomes are needed before recommendations about changing EI-related skills or job-satisfaction conditions can be treated as performance-enhancing interventions.

## 5. Conclusions

In this sample of 134 medical secretaries, emotional intelligence statistically accounted for part of the cross-sectional association between job satisfaction and self-reported job performance. The bootstrap indirect association was statistically supported (unstandardized ab = 0.114; 95% CI: 0.042–0.204; standardized ab = 0.172). After emotional intelligence was included in the model, the conditional direct association was not statistically significant (β = 0.103; *p* = 0.187), but this does not by itself establish causal mediation. The model explained 30.0% of the variance in self-reported performance, leaving 70.0% unexplained.

The reverse EI → job satisfaction → performance indirect association was not statistically supported in a post hoc sensitivity analysis. However, the cross-sectional design cannot establish temporal order or causality, and a common-antecedent explanation remains possible. The findings should therefore be generalized cautiously beyond the 134 medical secretaries in two public hospitals. Longitudinal, multi-source, multicenter studies using independently validated or objective performance measures are needed. Overall, job satisfaction and emotional intelligence appear to be jointly relevant to self-reported performance in this sample, but the findings should not be used alone for causal claims or individual personnel decisions.

## Figures and Tables

**Table 1:** Characteristics of the medical secretaries (*N* = 134).

Variable	Category	*n*	%
Age	Under 30 years	75	56.0
	30 years and over	59	44.0
Gender	Male	89	66.4
	Female	45	33.6
Marital status	Married	70	52.2
	Single	64	47.8
Education level	High school or below	24	17.9
	Associate degree	84	62.7
	Bachelor’s degree	26	19.4
Work experience	≤4 years	86	64.2
	5–13 years	32	23.9
	≥14 years	16	11.9

**Table 2:** Comparison of mean scale scores by characteristics of the medical secretaries.

Characteristics	EIS, M ± SD	JPS, M ± SD	JSS, M ± SD
Age			
Under 30 years	58.40 ± 13.45	16.39 ± 3.68	15.75 ± 5.44
30 years and over	61.29 ± 11.69	16.76 ± 3.03	17.76 ± 4.47
*t* (*p*)	−1.31 (0.194)	−0.63 (0.527)	−2.30 (0.023)
Gender			
Male	57.63 ± 13.82	16.01 ± 3.69	16.73 ± 4.92
Female	63.71 ± 9.14	17.62 ± 2.44	16.44 ± 5.54
*t* (*p*)	−2.67 (0.009)	−2.65 (0.009)	0.30 (0.761)
Marital status			
Married	60.17 ± 12.98	16.64 ± 3.73	17.11 ± 4.43
Single	59.13 ± 12.55	16.45 ± 3.03	16.11 ± 5.77
*t* (*p*)	0.47 (0.637)	0.32 (0.748)	1.14 (0.258)
Education level			
High school or below	52.63 ± 16.58	16.92 ± 3.28	16.58 ± 6.08
Associate degree	60.36 ± 12.03	16.37 ± 3.58	16.42 ± 5.05
Bachelor’s degree	63.96 ± 7.86	16.81 ± 2.97	17.38 ± 4.46
*F* (*p*)	5.63 (0.004) ^1^	0.33 (0.720)	0.35 (0.703)
Work experience			
≤4 years	58.91 ± 13.59	16.48 ± 3.72	16.09 ± 5.55
5–13 years	61.72 ± 10.28	16.44 ± 2.54	17.41 ± 4.21
≥14 years	59.69 ± 12.71	17.19 ± 3.21	18.00 ± 3.98
*F* (*p*)	0.56 (0.570)	0.32 (0.730)	1.42 (0.244)

Note: For the total sample, the means are EIS = 59.67 ± 12.74, JPS = 16.55 ± 3.40, and JSS = 16.63 ± 5.12. ^1^ Bonferroni-corrected pairwise comparisons for the education ANOVA: bachelor’s > high school or below; associate > high school or below. The table reports nominal *p* values for the 15 exploratory omnibus demographic comparisons; a post hoc Holm correction across these tests yielded no adjusted *p* < 0.05. EIS: Emotional Intelligence Scale; JPS: Job Performance Scale; JSS: Job Satisfaction Scale; M: mean; SD: standard deviation.

**Table 3:** Correlations among JSS, JPS, and EIS total scores (*N* = 134).

Variables	JSS	JPS
JPS	0.275 **	—
EIS	0.341 **	0.539 **

** *p* < 0.01.

**Table 4:** Regression models for the cross-sectional indirect association of job satisfaction with self-reported job performance through emotional intelligence.

Model	Path	B	SE	95% CI (B)	β	t	*p*	R^2^	Adj. R^2^
1	JSS → JPS	0.183	0.056	0.073; 0.293	0.275	3.285	0.001	0.076	0.069
2	JSS → EIS	0.849	0.204	0.449; 1.249	0.341	4.168	<0.001	0.116	0.110
3	JSS → JPS	0.069	0.052	−0.033; 0.171	0.103	1.326	0.187	0.300	0.289
	EIS → JPS	0.134	0.021	0.093; 0.175	0.504	6.477	<0.001		

Indirect association (JSS → EIS → JPS): ab = 0.114; 95% bootstrap CI [0.042; 0.204]; standardized indirect association = 0.172; 10,000 resamples. CI: confidence interval.

## Data Availability

The data presented in this study are available on request from the corresponding author. The data are not publicly available due to ethical restrictions.

## Abbreviations

The following abbreviations are used in this manuscript:

EIS/WLEIS	(Wong and Law) Emotional Intelligence Scale
JSS	Job Satisfaction Scale
JPS	Job Performance Scale (self-reported performance in this study)
ANOVA	Analysis of variance
CI	Confidence interval
SD	Standard deviation
SE	Standard error
